# Prevalence of delayed antiretroviral therapy initiation among people living with HIV: A systematic review and meta-analysis

**DOI:** 10.1371/journal.pone.0286476

**Published:** 2023-10-24

**Authors:** Yan Tao, Xueling Xiao, Ci Zhang, Ying Xie, Honghong Wang

**Affiliations:** Xiangya Nursing School, Central South University, Changsha, Hunan Province, People’s Republic of China; World Health Organization, SWITZERLAND

## Abstract

**Objective:**

HIV continues to be a global challenge. Key recommendations for HIV prevention and treatment are presented on rapid antiretroviral therapy (ART) initiation. However, several studies showed a high prevalence of delayed ART initiation. The aim of this systematic review and meta-analysis was to assess the prevalence of delayed ART initiation among HIV-infected patients globally.

**Methods:**

This review summarised eligible studies conducted between January 2015 and August 2022 on the prevalence of delayed ART initiation in HIV-infected adults (age ≥ 15). Relevant studies were systematic searched through PubMed/Medline, EMBASE, Web of Science, China National Knowledge Infrastructure, Wanfang, and Chongqing VIP databases. Random-effects models were used to calculate pooled prevalence estimates. The heterogeneity was evaluated using Cochran’s Q test and *I*^2^ statistics. Moreover, potential sources of heterogeneity were explored using univariate subgroup analysis.

**Results:**

Data on the prevalence of delayed ART initiation was pooled across 29 studies involving 34,937 participants from 15 countries. The overall pooled prevalence of delayed ART initiation was 36.1% [95% confidence interval (CI), 29.7–42.5%]. In subgroup analysis, the estimated pooled prevalence decreased with age. By sex, the prevalence was higher among male patients (39.3%, 95% CI: 32.2–46.4%) than female (36.5%, 95% CI: 26.9–50.7%). Patients with high CD4 cell count were more likely to delay ART initiation than those with low CD4 cell count (>500cells/mm^3^: 40.3%; 201-500cells/mm^3^: 33.4%; and ≤200cells/mm^3^: 25.3%).

**Conclusions:**

Our systematic review and meta-analysis identified a high prevalence of delayed ART initiation. The prolonged time interval between diagnosis and treatment is a prevalent and unaddressed problem that should spur initiatives from countries globally. Further research is urgently needed to identify effective strategies for promoting the early ART initiation.

## Introduction

HIV remains the leading cause of death and disability worldwide, endangering the health of the population and increasing the economic cost to health systems. By the end of 2021, 38.4 million people globally were infected with HIV, and 40.1 million people have died from AIDS-related diseases since the beginning of the epidemic [1]. Antiretroviral therapy (ART) is highly effective in slowing the progression of HIV to AIDS, reducing AIDS-related mortality and morbidity [2, 3], and preventing further HIV transmission, as achieving undetectable virus means nontransmissible virus [4, 5]; thus, timely initiation and full coverage of ART is necessary. However, only 87% of those who know their status have initiated ART, which is far from achieving the second ‘95’ of the UNAIDS ‘95-95-95’ target by 2025 [1, 6].

Early initiation of treatment after diagnosis maximizes the benefits of ART at the individual and population levels. Randomized controlled trials in Uganda and South Africa demonstrated the feasibility and improvement in terms of treatment coverage and retention rates over three months with same-day initiation of ART, compared to the standard initiation group [7, 8]. The HPTN 052 trial in nine African countries clearly showed that early initiation of ART with CD4 counts between 350 and 550 cells /mm^3^ facilitated the faster achievement of viral load suppression and reduced HIV transmission by approximately 96% compared to late ART initiation with CD4 count < 250 cells /mm^3^ [9]. Observational studies conducted in South Africa [10], Eswatini [11] and Ethiopia [12], which compared early treatment with standard care, reported similar results. Moreover, if ART is initiated immediately, the cost of hospitalization may be minimized in resource-poor and HIV-prone prevalence countries [13, 14]. As a result of this evidence, the World Health Organization (WHO) recommended in 2015 that all HIV-infected patients should start ART once they are diagnosed with HIV, regardless of stage of disease and CD4 cell count (known as “treat all”). There is no reason to delay treatment decisions based on CD4 cell threshold [15, 16].

However, despite knowing the benefits of early treatment and the expanding ART eligibility criteria, delayed initiation of ART persists and some countries face challenges in reaching the ‘95-95-95’ targets [1]. A retrospective study reviewing the rate of ART initiation in HIV-infected ART-naive women in Uganda, revealed delayed treatment rates of up to 72% [17]. Wei et al. analyzed the timeliness of ART initiation in newly reported HIV-infected patients in Hunan Province, China, from 2014 to 2018, suggesting that 58% of patients did not initiate ART within one month of diagnosis [18]. A pilot randomized controlled trial from 2015 to 2016 indicated that 67% of HIV-infected adults were linked to care and 42% initiated ART within three months of referral for treatment [19]. A systematic review of HIV care retention has shown that the longer lag time between testing and treatment, the higher the rate of patients lost to ART care [20]. Taking these studies together, reliable estimates of delayed ART initiation after the introduction of the “treat all” strategy and understanding various potential related factors are essential for informing ongoing and future intervention strategies to effectively improve coverage of ART.

In general, there is no unified, clear, evidence-based definition of delayed initiation of treatment. Various delayed ART initiation for time to ART initiation have been used in the studies, such as time from diagnosis to ART initiation, time from first clinical visit to ART initiation [21], time from eligibility to ART initiation [22], and time from enrolment in HIV care to ART initiation [23]. Time from HIV diagnosis to commencement of ART was proposed as a more feasible and sensitive indicator of ART uptake [24]. In our study, delayed ART initiation was defined as failure to initiate ART within 30 days of confirmation of HIV diagnosis, as previous studies have found that one month was sufficient for most patients to complete the steps needed to start treatment under routine care [25, 26].

To the best of our knowledge, there are no current or ongoing systematic reviews of the prevalence of delayed ART initiation, although sufficient data exist to make pooled estimates and to explore the source of heterogeneity between studies through meta-analysis. To fill this critical gap, we conducted this study to estimate the global prevalence of delayed ART initiation and identify social and epidemiological factors related to delayed ART initiation in published observational studies in HIV-infected adults, by a systematic review and meta-analysis.

## Materials and methods

### Search strategy

We searched three English language electronic databases, PubMed/Medline, Web of Science, and Embase, and three Chinese language Databases, CNKI (China National Knowledge Infrastructure), Wanfang, and Chongqing VIP databases. Our comprehensive search strategies combined terms and variants of HIV (“human immunodeficiency virus”, “acquired immunodeficiency syndrome”), treatment (therapeutics, “antiretroviral therapy, highly active”), and delay (Time-to-Treatment, defer, same-day, early, instant, immediate, rapid, quick) using both controlled vocabularies and free text words. Search queries were optimized to fit the specific feature of each database (S1 File). The electronic database search was supplemented by scanning the reference list of included articles, previous reviews and published meta-analysis. All study steps of this systematic review and meta-analysis were conducted in accordance with the Preferred Reporting Items for Systematic Reviews and Meta-Analysis (PRISMA) guidelines (S1 Table) [27].

### Inclusion and exclusion criteria

Studies complying with the following inclusion criteria were included: (1) Studies published in English or Chinese between January 2015 and August 2022; (2) HIV-infected adults (≥15Y); (3) Studies reported time to ART initiation (namely, time from diagnosis to ART initiation) and delayed initiation prevalence or gave sufficient information to calculate this estimate. We excluded case reports, study protocols, commentaries, correspondence, letters, reviews or systematic reviews and studies carried out with pregnant women or people with serious, opportunistic infections due to the clinical management of these patients being complex.

### Selection of studies

All searched articles from different databases were exported to the Endnote reference manager, and duplicates were removed. Then, two authors (Tao and Xie) independently evaluated the potentially relevant articles by checking the titles and abstracts. Those articles that did not meet the inclusion criteria were removed, and the full texts of the remaining articles were retrieved based on the inclusion and exclusion criteria. Any discrepancies that arose during the selection process were resolved by the adjudicator (Xiao).

### Data extraction and quality assessment

The following elements was extracted from included articles, with use of a preconceived data abstraction form: (1) Characteristics of the study: author name, publication year, study year, study design, country, country income and continent; (2) Characteristics of the sample: sample size, age, gender; (3) HIV-related data: CD4 cell count, the time between HIV diagnosis and ART initiation, the number of participants who delayed ART initiation.

We assessed the methodological quality of included studies using the Newcastle–Ottawa Scale [28]. Though this tool, risk of bias was assessed across the domains of selection, comparability and outcome for the risk of bias revision, and high-quality articles were determined when the score of the scale is five and above out of nine scales. Two authors (Tao and Xie) independently assessed study quality, and disagreements were discussed until a consensus was reached.

### Statistical analysis

Statistical analysis was performed using Stata 15. The prevalence estimates of delayed initiation of ART and their 95% confidence interval (CI) were calculated and pooled by time elapsed from HIV diagnosis to ART initiation using a random effect analysis model, accounting for the expected between-study significant heterogeneity [29]. Q-tests and *I*^2^ statistics were used to assess the level of heterogeneity among studies [30]. The *I*^2^ heterogeneity was categorized as follows: low (25–50%), moderate (50–75%) and high (>75%). Subgroup analysis was undertaken to investigate source of between-study heterogeneity, in terms of country, continent, study year, study design, sample size, age group, gender, CD4 cell count. Sensitivity analysis was carried out by serially omitting each study to assess the influence of individual studies on the robustness of the pooled estimate. Furthermore, publication bias was evaluated though visual inspection of the funnel plot and Egger’s tests [31].

## Results

### Study results and characteristics

**Fig 1:**
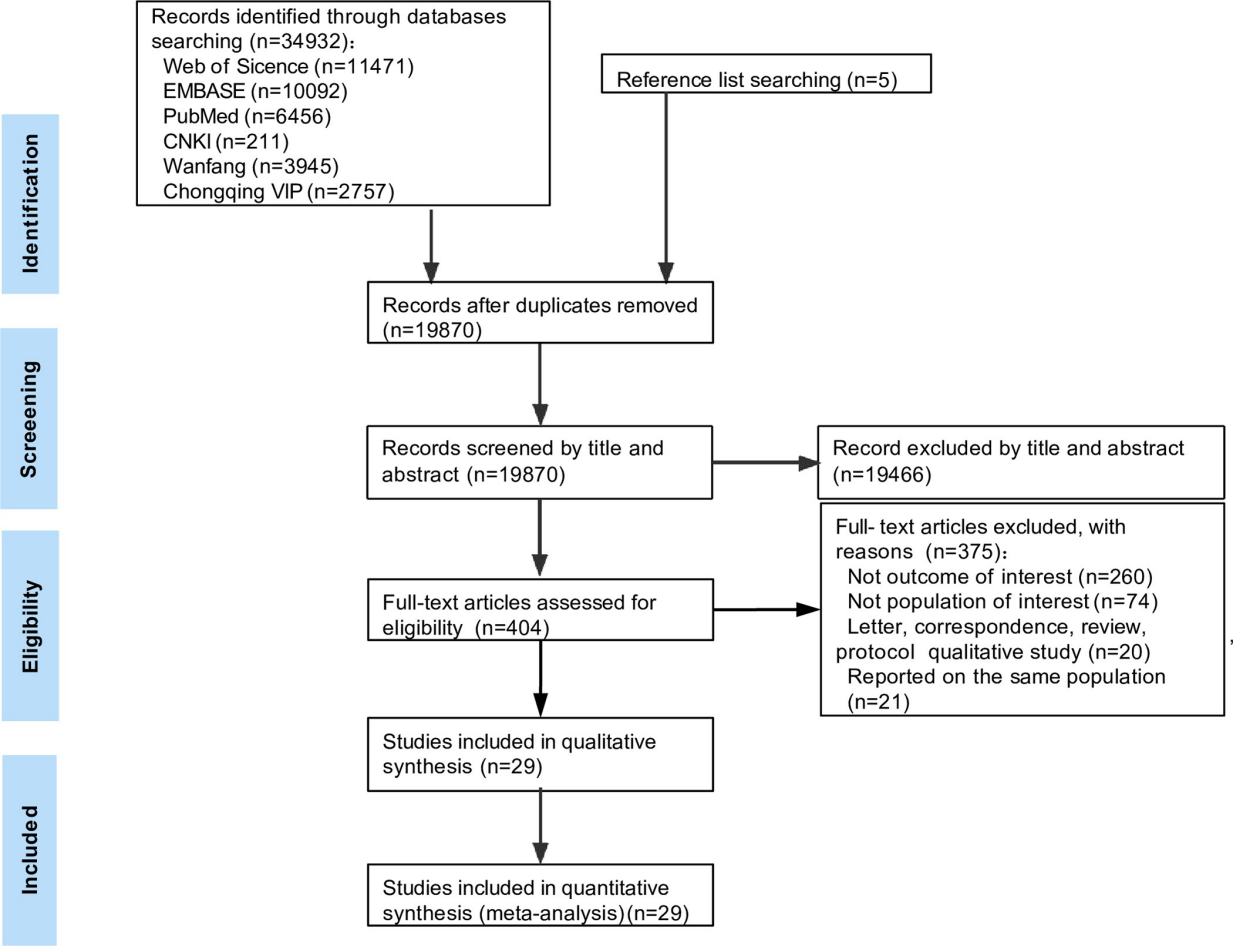
Flow diagram of the studies included in the meta-analysis on delayed ART treatment.

The PRISMA flow diagram outlines the search strategy to identify the articles (Fig 1). In total, 34937 publications were identified, of which 34533 were excluded as duplicated or irrelevant according to title and abstract. Full-text assessment was undertaken for leaving 404 potentially articles. After a detailed full-text review, 29 eligible articles [32–60] were included in the analysis.

Number of participants ranged from 244 in a Chinese cohort [49] to 34528 in an African cohort [43], and a total of 121142 participants across 15 countries participated in the 29 included studies. More than half of the studies (n = 16) [37, 46–60] were conducted in Asia, followed by Africa (n = 9), Europe (n = 3) [32, 35, 40] and North America (n = 1) [39]. The target population included ART-initiated patients and HIV-positive patients including patients who did not start treatment. One study only provided prevalence in patients aged from 15 to 19 years [46]. Seven studies reported the mean time to initiate ART after HIV confirmation, ranging from five to 40 days [32, 35, 36, 46, 47, 49, 52]. In addition, 18 studies used a cohort [32–37, 39, 41–45, 48–52, 54] design, 10 studies used a cross-sectional design [40, 46, 47, 53, 55–60], and one study [38] used quasi-experimental study. We evaluated the quality of 28 of 29 studies. One meeting abstract cannot be accessed without full text [34]. The quality of 28 studies ranged from three to eight. A detailed description of the characteristics of all enrolled studies is displayed in Table 1.

**Table 1:** Summary of included studies on prevalence of delayed ART initiation.

Author	Year	Country	Continent	Country income	Study design	Population	Age, mean (IQR)(y)	Male	Mean time to ART initiation (d)	Sample size	Event	Quality score
**Lee et al. [32]**	2019	UK	Europe	High income	Retrospective cohort	HIV-positive	≥18, 37 (31–47)	213	29d (R:0-514d)	292	165	7
**Mshweshwe-Pakela et al. [33]**	2020	South Africa	Africa	Lower-middle income	Retrospective cohort	HIV-positive	≥18, 32 (27–39)	298	NR	826	172	7
**Ross et al. [34]**	2020	Rwanda	Africa	Lower-middle income	Prospective cohort	HIV-positive	≥15	NR	NR	1971	458	—^a^
**D’Arminio et al. [35]**	2020	Italy	Europe	High income	Prospective cohort	ART-initiated	≥18, 38 (29–47)	1038	40d (IQR:21-73d)	1247	783	7
**Onoya et al. [36]**	2020	South Africa	Africa	Lower-middle income	Prospective cohort	HIV-positive	≥18	NR	R: 5-8d	883	197	6
**Teeraananchai et al. [37]**	2020	Thai	Asia	Upper-middle income	Retrospective cohort	HIV-positive	R: 15–24	22075	NR	29782	14174	8
**Lebelonyane et al. [38]**	2020	Botswana	Africa	Upper-middle income	Quasi-experimental	HIV-positive	33 (26–41)	361	NR	800	122	7
**Bacon et al. [39]**	2021	USA	North America	High income	Retrospective cohort	HIV-positive	≥30	NR	R: 6-37d	915	500	7
**Kaide et al. [40]**	2021	UK	Europe	High income	Cross-sectional	HIV-positive	≥16	1704	NR	2281	1182	3
**Onoya et al. [41]**	2021	South Africa	Africa	Lower-middle income	Prospective cohort	HIV-positive	≥18	NR	NR	266	95	6
**Dah et al. [42]**	2021	Cote d’Ivoire, Mali, Togo, Burkina Faso	Africa	Lower-middle income low income, low income, low income	Prospective cohort	HIV-positive, MSM	24.0 (21.2–27.7)	350	5d (IQR: 1-13d)	350	52	6
**Mody et al. [43]**	2021	Zambia	Africa	Lower-middle income	Retrospective cohort	HIV-positive	≥18	13116	NR	34528	4489	8
**Beesham et al. [44]**	2022	Eswatini,Kenya, South Africa, Zambia	Africa	Upper-middle income, Lower-middle income, Upper-middle income Lower-middle income,	Retrospective cohort	HIV-positive	16–35	0	NR	304	176	7
**MacKellar et al. [45]**	2022	Eswatini	Africa	Upper-middle income	Retrospective cohort	HIV-positive	≥15	319	306d (IQR: 8d-unknown)	769	505	7
**Xu et al. [46]**	2019	China	Asia	Upper-middle income	Cross-sectional	ART-initiated, sexual transmission	R: 15–19	NR	29d (IQR:13-85d)	4990	2462	4
**Yu et al. [47]**	2019	China	Asia	Upper-middle income	Cross-sectional	ART-initiated	≥15	NR	38d (IQR:16-186d)	5191	2348	4
**Ma et al. [48]**	2019	China	Asia	Upper-middle income	Retrospective cohort	HIV-positive, Heterosexual transmission	≥18	NR	NR	4409	1157	7
**Yuan et al. [49]**	2020	China	Asia	Upper-middle income	Retrospective cohort	ART-initiated	≥15, 36 (27,46)	213	15d (IQR:11-26d)	244	49	5
**He et al. [50]**	2020	China	Asia	Upper-middle income	Retrospective cohort	ART-initiated	≥18	NR	NR	1504	334	7
**Zhang et al. [51]**	2020	China	Asia	Upper-middle income	Retrospective cohort	HIV-positive	≥16, 46.52±16.09	335	NR	406	185	7
**Nong et al. [52]**	2021	China	Asia	Upper-middle income	Retrospective cohort	HIV-positive	≥30	NR	R: 11-12d	3976	1377	7
**Wu et al. [53]**	2021	China	Asia	Upper-middle income	Cross-sectional	ART-initiated	≥18	NR	17d	3528	864	6
**Yu et al. [54]**	2021	China	Asia	Upper-middle income	Retrospective cohort	HIV-positive	32.7±11.4	2197	NB	2197	812	7
**Feng et al. [55]**	2021	China	Asia	Upper-middle income	Cross-sectional	ART-initiated	≥15	1819	1.35m (IQR: 0.53–9.55m)	2054	971	7
**Zhu et al. [56]**	2021	China	Asia	Upper-middle income	Cross-sectional	ART-initiated	36.05±12.51	6813	NB	7425	2967	7
**Yang et al. [57]**	2022	China	Asia	Upper-middle income	Cross-sectional	HIV-positive	R: 16–92, 54.79±16.56	744	NB	1026	402	6
**Tian et al. [58]**	2022	China	Asia	Upper-middle income	Cross-sectional	ART-initiated	≥18	2568	NB	3859	1258	6
**Guo et al. [59]**	2022	China	Asia	Upper-middle income	Cross-sectional	HIV-positive	≥15	3121	NB	3281	926	6
**Yang et al. [60]**	2022	China	Asia	Upper-middle income	Cross-sectional	HIV-positive	R: 30–90	NR	NB	1065	416	6

Abbreviations: ART = Antiretroviral Therapy; HIV = Human Immunodeficiency Virus; d = days; m = months, NR = Not Report; IQR = Interquartile Range; R = Range

^a^The meeting abstract cannot be accessed without full text

### Prevalence of delayed initiation of ART

**Fig 2:**
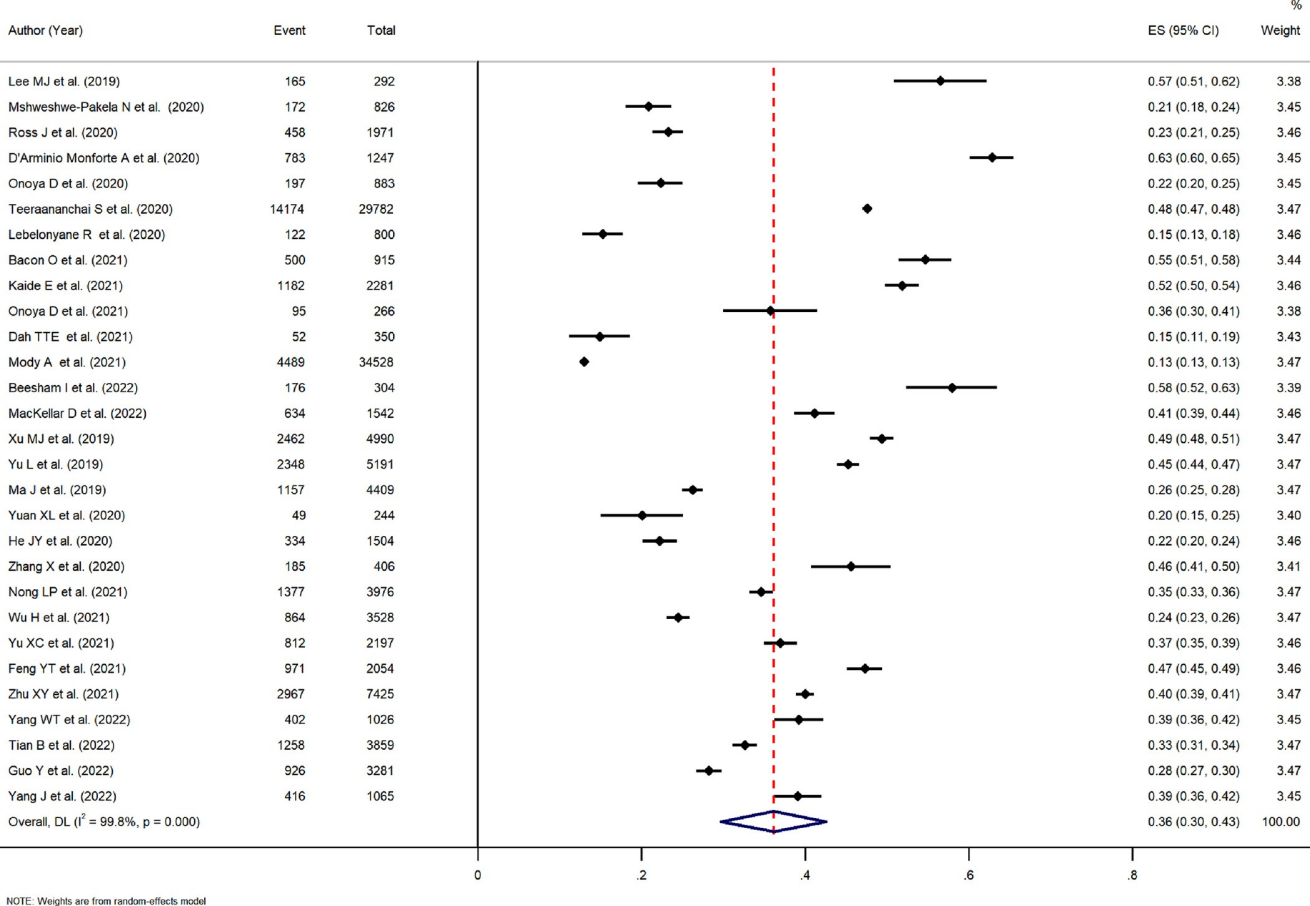
Estimated prevalence of delayed ART initiation. NOTE: Weights are from random-effects model.

Analyzing the global prevalence of delayed initiation of ART among patients using the random-effects model, we found the pooled prevalence of delayed ART initiation was 36.1% (95% CI: 29.7–42.5%; *I*^2^ = 99.8, *p*<0.001). Fig 2 shows the forest plot of the 29 studies reporting the prevalence of delayed treatment.

### Subgroup analysis

Subgroup analyses were conducted to compare the prevalence of delayed ART initiation by regions, research design, age, gender, and CD 4 cell count (Table 2).

**Table 2:** Subgroup analysis of prevalence of delayed ART initiation.

Category	Subgroup	No of study	Prevalence (95% CI) (%)	Sample	*I*^2^ (%)	*P*	*P* (Egger’s test)	Between-group difference
**Total**		29	36.1 (29.7–42.5)	121142	99.8	<0.001	0.027	
**Continent**	Asia	16	36.2 (31.4–41.0)	74937	99.4	<0.001	0.056	
	Africa	9	26.9 (19.4–34.4)	41470	99.2	<0.001	0.013	
	Europe	3	57.0 (49.1–65.0)	3820	95.1	<0.001	0.778	
	North America	1	54.6 (51.4–57.9)	915	-	-	-	
								*p*<0.001
**Country**	China	15	39.1 (36.1–42.0)	45155	99.1	<0.001	0.890	
	Others	14	41.0 (38.7–43.6)	75987	99.9	<0.001	0.209	
								*p* = 0.695
**Country income**	High income	4	56.4 (50.8–62.1)	4735	92.7	<0.001	0.709	
	Upper-middle income	18	35.5 (30.8–40.1)	77297	99.5	<0.001	0.029	
	Lower-middle income	5	22.7 (16.1–29.3)	38474	98.3	<0.001	0.010	
								*p*<0.001
**Study design**	Cohort	18	35.3 (26.2–44.4)	85642	99.9	<0.001	0.153	
	Cross-sectional	10	39.7 (33.9–45.6)	34700	99.2	<0.001	0.727	
	Quasi-experimental	1	15.3 (12.8–17.7)	800	-	-	-	
								*p*<0.001
**Sample size**	<1000	10	45.6 (23.8–44.8)	5286	98.8	<0.001	0.092	
	1000–3000	9	40.4 (31.7–49.1)	14887	99.2	<0.001	0.269	
	>3000	10	34.1 (22.8–45.5)	100969	99.8	<0.001	0.125	
								*p*<0.001
**Sex**	Male	9	39.3 (32.2–46.4)	15109	98.6	<0.001	0.863	
	Female	8	36.5 (26.9–50.7)	2237	97.2	<0.001	0.257	
								*p* = 0.036
**Age-specific group (Y)**	15–29	6	43.3 (32.6–54.0)	7590	98.4	<0.001	0.629	
	30–49	4	39.8 (25.1–54.5)	3721	98.8	<0.001	0.432	
	50-	5	38.4 (33.8–43.0)	4577	98.2	<0.001	0.569	
								*p* = 0.041
**CD4 cell count (cells/mm** ^**3**^ **)**	≤200	6	25.3 (18.7–31.9)	2410	93.1	<0.001	0.175	
	201–500	3	33.4 (17.0–49.8)	1864	97.8	<0.001	0.984	
	>500	3	40.3 (14.0–66.5)	659	98.1	<0.001	0.673	
								*p*<0.001

Prevalence estimates from studies performed in China (39.1%, 95% CI: 36.1–42.0%) was lower than those performed outside China (41.0%, 95% CI: 38.7–43.6%). The pooled prevalence in high-income countries (56.4%, 95% CI: 50.8–62.1%) was higher than that in upper-middle income countries (35.5%, 95% CI: 30.8–40.1%) and in lower-middle income countries (22.7%. 95% CI: 16.1–29.3%). We further stratified studies by continent. The pooled estimates were highest for studies conducted in Europe. The estimated pooled prevalence among the studies that used cross-sectional design (37.5%, 95% CI: 31.3–43.7%) was higher than those studies that used cohort design (35.3%, 95% CI: 26.2–44.4%). In addition, the pooled prevalence decreased with age. The prevalence was the highest in the 15–29 age group compared to in the 30–49 age group and the lowest in patients aged 50 or over, with pooled prevalence 43.3%, 39.8%, and 38.4%, respectively. When the prevalence was stratified by gender, we found that male population (39.3%, 95% CI: 32.2–46.4%) had a slightly higher prevalence than female population (36.5%, 95% CI: 26.9–50.7%). With the CD4 cell count, the pooled estimates showed an upward trend. Pooled prevalence estimates for patients with CD4 cell count under 200 cells/mm^3^, between 200-500cells/mm^3^ and over 500 cells/mm^3^ was 25.3%, 33.4% and 40.3%, respectively.

### Sensitivity analysis

We performed a sensitivity analysis using the random-effects model to identify the effect of individual studies on the overall meta-analysis. No significant changes in the overall pooled prevalence for delated ART initiation were found on the removal of a single study (S1 Fig).

### Publication bias

**Fig 3:**
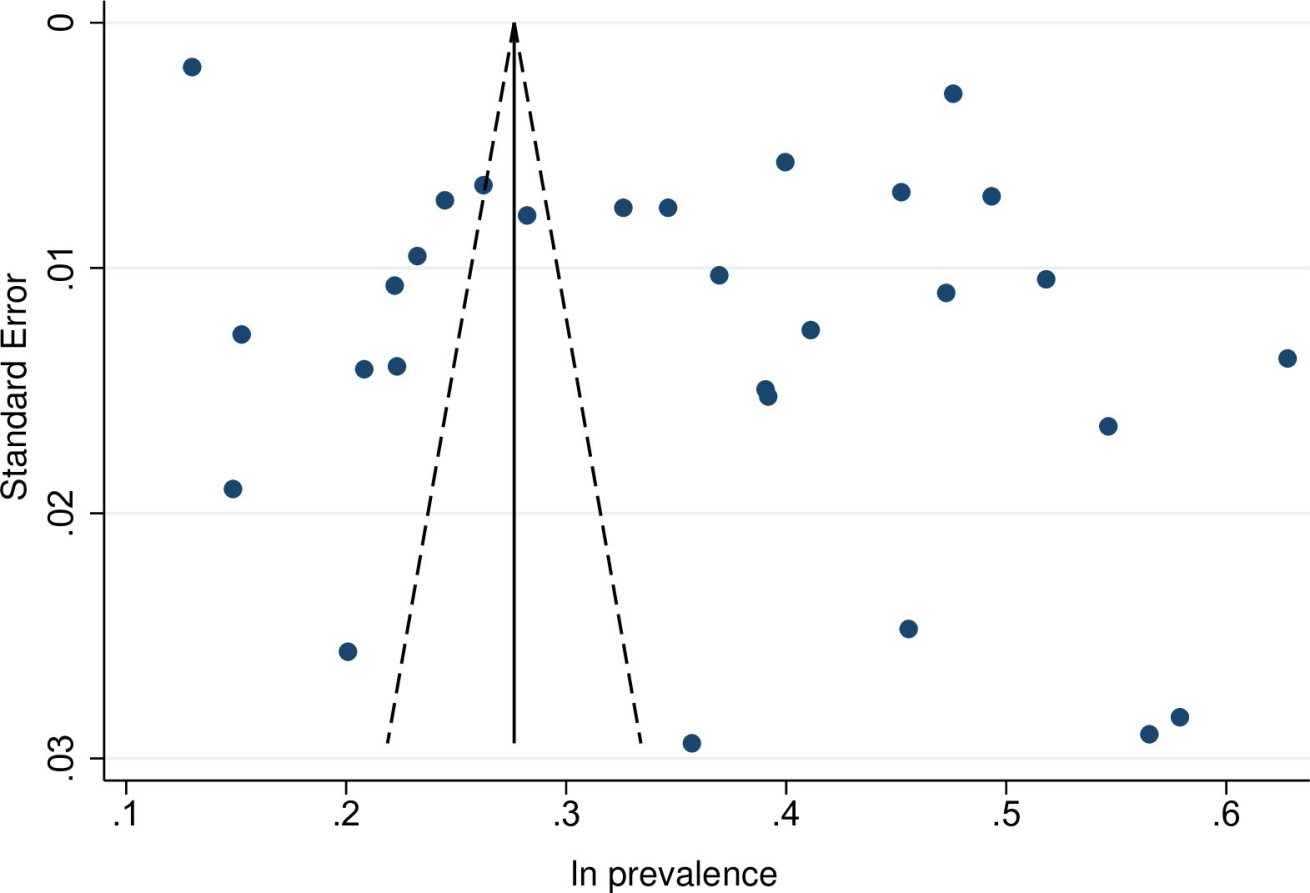
The funnel plot of studies reporting the prevalence of delayed ART initiation.

Visual inspection of the funnel plot of studies reporting on the delayed treatment revealed significant asymmetry (Fig 3). The egger’s test showed evidence of significant publish bias (*P* = 0.027) which further support the finding of the funnel plot.

## Discussion

The current study is the first meta-analysis using a comprehensive search strategy to explore the global prevalence of delayed ART initiation among HIV-infected patients. The studies included in this review showed a high prevalence of delayed ART initiation. Our study revealed that the pooled prevalence was 38.0%, lower than the pre-2015 delayed initiation rate ranging from 41%-85% [61–64]. A previous systematic review of six studies conducted in sub-Saharan Africa in 2012 by Mugglin et al. found that the median time of the whole period from HIV diagnosis to ART initiation ranged from a few weeks to more than six months [65]. In our study, the median time from HIV diagnosis to ART initiation varied from five days to 40 days, which is still a gap from the WHO recommendation of starting treatment within seven days or on the same day of confirming HIV diagnosis [66].

Although the WHO guidelines encourage early initiation of ART and HIV treatment has proven to be effective, the decision to start treatment takes into account various factors. It is increasingly recognized that, earlier ART initiation is associated with a low incidence of severe HIV morbidity, disease progression, better immune recovery and longer survival time [67]. Despite this, some patients may opt to delay initiation of ART due to concerns about side effects, pill interaction and financial constraints. This is particularly true for patients who have non-AIDS-defining conditions and serious, opportunistic infections that fall outside the scope of this study [68]. In addition, different countries have recommended ART initiation at varying levels of CD4 count because of medical resources and economic concerns [67, 69].

In this systematic review, we identified studies from 15 countries. Surprisingly, patients in high-income countries delayed treatment at a much higher rate than patients in upper- and lower-middle income countries. This might be explained by the fact that implementing “treat all” is rapidly expanding in upper- and lower-middle income countries, 82 of which have officially adopted the recommendation to provide universal HIV treatment to HIV-infected individuals [6]. In addition, many effective interventions, including simplified ART initiation procedures [8, 70], short message service (SMS) health messages [71], and grassroots prevention and control service model which integrated the resources of the health and medical service network and accelerated HIV treatment services [52], have been implemented in upper- and lower-middle income countries, which accelerated the initiation of treatment. The relationship between national income levels and late treatment initiation needs to be further explored.

Our systematic review found that delayed initiation of ART was more common in male patients than in females, a finding that is in line with the previous study conducted in 6 sub-Saharan African countries showing that men had a significantly higher risk of delaying treatment after enrollment in HIV care than women [72]. Hibbard et al. also reported that females use HIV health services more often than males [73]. However, gender inequalities and gender-based violence persist, increasing women’s blocks access to services. Previous studies revealed a positive association between use of ART among women and sexual and physical partner violence [74, 75]. In 2021, UNAIDS Board adopted the new Global AIDS Strategy 2021–2026, End Inequalities, End AIDS, which propels the process of ending AIDS [76]. Strategies and attention on tackling delayed initiation of ART should thus be focused equally on both genders.

We discovered that patients younger than 30 years of age were more likely to delay initiation of treatment than patients older than 50 (47.3% VS 43.3%), which is consistent with other studies conducted among general HIV-infected patients [77, 78]. A possible reason for the age difference is that since HIV requires lifetime treatment, patients who start treatment at a younger age will get a longer duration of therapy, and long-term medicine would be a serious burden. Financial stress has been identified as a main barrier to initiating and adhering to HIV treatment among young adults [79]. Substantial subsidies for ART drugs, as well as more social benefits for young adults, are expected to increase their affordability, especially in terms of paying out-of-pocket for prescription ART drugs, so that they can rapidly start HIV treatment and achieve higher ART coverage [80].

Our study also found that a higher CD4 cell count was related to the late onset of ART. Although the implementation of the new WHO guidelines on HIV treatment, regardless of CD4 count, was effective for timely initiation of ART, especially for patients who are unavailable to services and have a high probability of delayed or non-initiation of treatment after diagnosis [81, 82], a high CD4 cell count remains a risk factor for timing initiation of treatment. This may be because many asymptomatic HIV-infected patients consider themselves to be healthy in the presence of high CD4 counts. As a result, they thought they could delay treatment until they develop HIV-related symptoms [83]. Previous studies have also confirmed that feeling healthy and the late appearance of HIV-related symptoms are independent factors for the late initiation of ART [84]. ART initiation counseling should highlight the benefit of early treatment and motivate them to participate in the care system. Additional support and other intervention measures to facilitate the care cascade over time were needed for those patients.

Given the current prevalence of delayed ART initiation, effective measures are needed to increase patient awareness of early treatment. Failure to initiate treatment rapidly after diagnosis hinders the effectiveness of ART as a prevention method for HIV mortality and morbidity, suggesting a great need to explore patient concerns about starting treatment and develop strategies to overcome them. Several studies have identified factors associated with delayed ART initiation, including structural and individual-level barriers [23, 85, 86]. The inconvenience of treatment as structural barriers, side effects, stigma, and lack of support as individual-level barriers were the most common concerns about ART initiation. Increased social support from close friends and family has been shown to be effective in facilitating ART initiation [87]. A systematic review suggested that home-based ART services as an alternative to a centralized medical care system were another effective way to deliver ART and increase linkage to care [88].

Despite the robust findings, our study has some limitations. First, some relevant literature may have been missed, since only English or Chinese articles were included, and grey literature was not searched. Additionally, most of data were derived from studies in China and South Africa, and the pooled estimates could not be well representative of the world. Further, some of the studies in our analysis were conducted in patients who initiated ART, and those not receiving treatment were not included in the study, which may lead to an underestimation of the rate of delayed ART initiation. Lastly, the observed heterogeneity was high in the overall analysis. Although subgroup analysis found some factors that affect the result, the extent to which we can explain observed heterogeneity is limited.

## Conclusions

Our systematic review revealed a high prevalence of delayed ART initiation. Comprehensive strategies and collaboration between countries and multilateral institutions to accelerate ART initiation have become increasingly critical, and the WHO recommendation supporting rapid initiation should be implemented. The prevalence of delayed treatment was higher in younger patients and those with high CD4 counts, suggesting more effective intervention strategies to promote early treatment should be provided to those high-risk patients.

## Supporting information

S1 File Datail of PubMed database search strategies. (DOCX)

S1 Table PRISMA 2020 checklist. (DOCX)

S1 Fig Sensitivity analysis. (TIF)
