# Supplementary material for: Prevalence of delayed antiretroviral therapy initiation among people living with HIV: A systematic review and meta-analysis
Source: PLoS One. 2023 Oct 24;18(10):e0286476. doi: 10.1371/journal.pone.0286476 (PMC10597480; doi:10.1371/journal.pone.0286476)
Supplement: S1 File — (DOCX) [file pone.0286476.s001.docx]

**S1 File. Datail of PubMed database search strategies.**

| **Search** | **Query** |
| --- | --- |
| #1 | Search: "HIV"[MeSH Terms] |
| #2 | Search: "HIV-1"[MeSH Terms] OR "HIV-1"[Title/Abstract] OR "HIV1"[Title/Abstract] |
| #3 | Search: "HIV-2"[MeSH Terms] OR "HIV-2"[Title/Abstract] OR "HIV2"[Title/Abstract] |
| #4 | Search: "human immunodeficiency virus"[Title/Abstract] OR "human immuno deficiency virus"[Title/Abstract] OR "human immune deficiency virus"[Title/Abstract] OR "human immune deficiency virus"[Title/Abstract] |
| #5 | #1 OR #2 OR #3OR #4 |
| #6 | Search: "HIV Infections"[MeSH Terms] OR "human immunodeficiency virus infection"[Title/Abstract] |
| #7 | Search: "Acquired Immunodeficiency Syndrome"[MeSH Terms] OR "acquired immuno deficiency syndrome"[Title/Abstract] OR "acquired immune deficiency syndrome"[Title/Abstract] OR "acquired-immune deficiency syndrome"[Title/Abstract] OR "hiv/aids"[Title/Abstract] |
| #8 | #5 OR #6 OR #7 |
| #9 | Search: "Therapeutics"[MeSH Terms] |
| #10 | Search: "antiretroviral"[Title/Abstract] OR "anti-retroviral"[Title/Abstract] OR "anti retroviral"[Title/Abstract] OR "anti virus"[Title/Abstract] OR ("anti acquired"[All Fields] AND "immuno-deficiency"[Title/Abstract]) OR "anti acquired immune deficiency"[Title/Abstract] OR "anti acquired immunodeficiency"[Title/Abstract] OR "anti acquired immune deficiency"[Title/Abstract] OR "anti-hiv"[Title/Abstract] |
| #11 | Search: "Anti-Retroviral Agents"[MeSH Terms] OR "Anti-Retroviral Agents"[Pharmacological Action] OR "Anti-Retroviral Agents"[Title/Abstract] |
| #12 | Search: "antiretroviral therapy, highly active"[MeSH Terms] OR "highly active antiretroviral therapy"[Title/Abstract] OR "highly active anti retroviral therapy"[Title/Abstract] OR "highly active antiretroviral treatment"[Title/Abstract] OR "ART"[Title/Abstract] OR "HAART"[Title/Abstract] OR "combination antiretroviral therapy"[Title/Abstract] OR "combined antiretroviral therapy"[Title/Abstract] OR "cART"[Title/Abstract] |
| #13 | #9 OR #10 OR #11 OR #12 |
| #14 | Search: (("Time-to-Treatment"[MeSH Terms] OR "delay*"[Title/Abstract] OR "same-day"[Title/Abstract] OR "defer*"[Title/Abstract] OR "early"[Title/Abstract] OR "instant"[Title/Abstract] OR "immediate"[Title/Abstract] OR "rapid"[Title/Abstract] OR "rapidly"[Title/Abstract] OR "quick"[Title/Abstract] OR "quickly"[Title/Abstract]) OR (timeliness[Title/Abstract])) AND (((((initiat*[Title/Abstract]) OR (treatment[Title/Abstract])) OR (therapy[Title/Abstract])) OR (ART[Title/Abstract])) OR (antiretroviral*[Title/Abstract])) |
| #15 | #8 AND #13 AND #14 |
| #16 | #8 AND #13 AND #14 Filters: Chinese, English, from 2015/1/1 - 2022/8/6 |

Abbreviations: ART, antiretroviral therapy; HIV, human immunodeficiency virus
