# Supplementary figures and images for: Prevalence of delayed antiretroviral therapy initiation among people living with HIV: A systematic review and meta-analysis
Source: PLoS One. 2023 Oct 24;18(10):e0286476. doi: 10.1371/journal.pone.0286476 (PMC10597480; doi:10.1371/journal.pone.0286476)

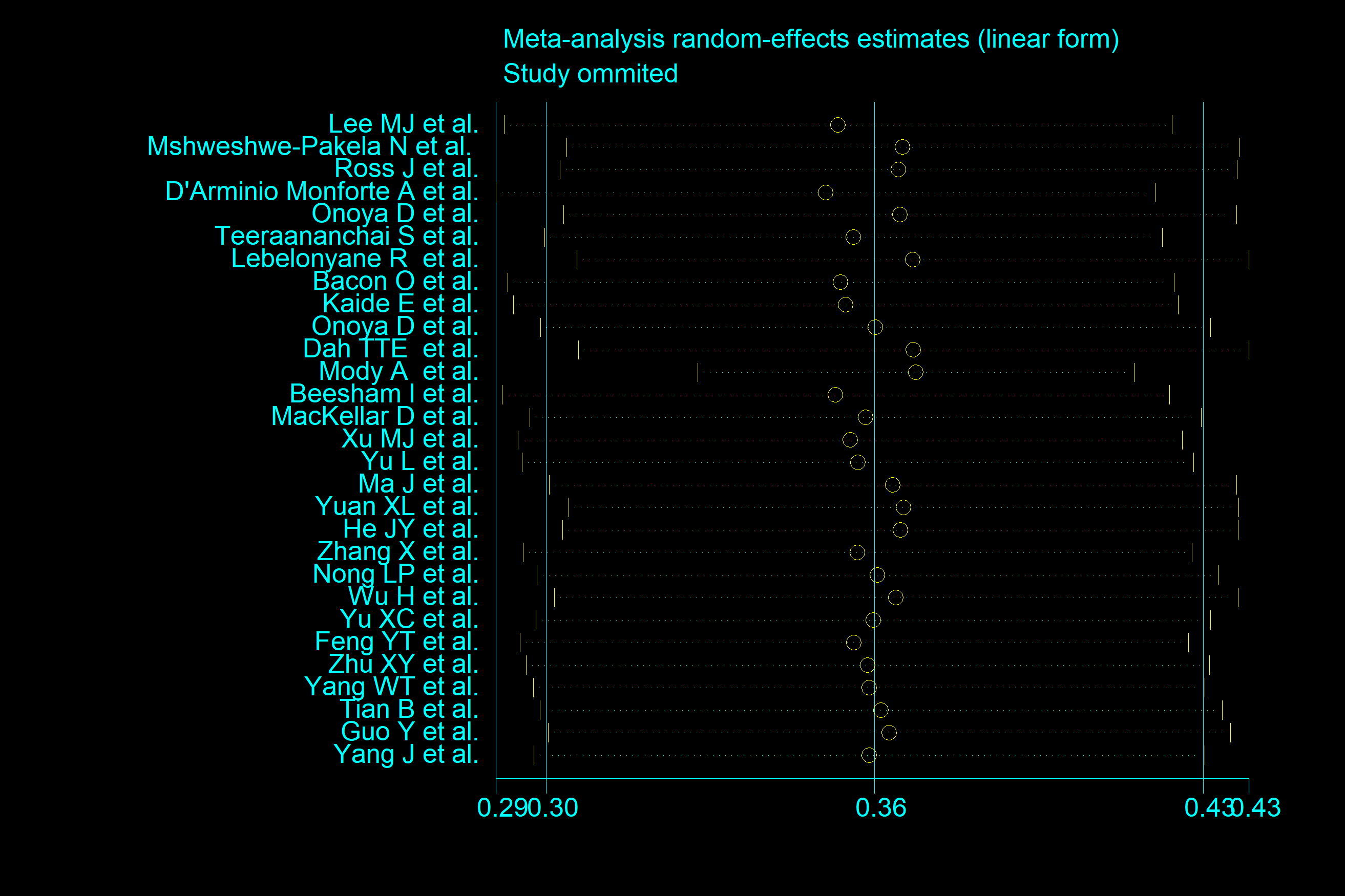

Supplement: S1 Fig — (TIF) [file pone.0286476.s003.tif]
