# Supplementary material for: Prevalence of delayed antiretroviral therapy initiation among people living with HIV: A systematic review and meta-analysis
Source: PLoS One. 2023 Oct 24;18(10):e0286476. doi: 10.1371/journal.pone.0286476 (PMC10597480; doi:10.1371/journal.pone.0286476)
Supplement: Attachment — Submitted filename: Response to reviwers.docx [file pone.0286476.s004.docx]

**XiangYa School of Nursing**

**Central South University**

Dr. Chuanyi Ning

Academic Editor

PLOS ONE

December 19, 2022

Re: Manuscript PONE-D-22-26544 “Prevalence of delayed antiretroviral therapy initiation among people living with HIV: a systematic review and meta-analysis”.

Dear Editor and Reviewers

Thank you for reviewing our manuscript PONE-D-22-26544 “Prevalence of delayed antiretroviral therapy initiation among people living with HIV: a systematic review and meta-analysis”. We found the comments very helpful in improving our manuscript. Below we list the reviewer comments and our responses. We had presented the updated changes in “italic”.

* * * * *

Comments of Reviewer 1:

Reviewer #1: Please consider the following comments:

1. The manuscript should be proofread carefully.

***Response:*** *Many thank. We have revised our manuscript carefully*.

2. Line 15: Please delete the working definition for delayed antiretroviral therapy

***Response:*** *Many thanks. We have deleted the definition (Line 15)*.

3. Line 21: Please provide both names of the database and platform (e.g. Pubmed/Medline)

***Response*:** *Many thanks for your comment. We have revised this part (Line 19, 88)*.

4. Line 23: Why the authors used both approaches for detecting heterogeneity?

***Response*:** *Many thanks for your question. We used Cochran’s Q test and I² statistics to detect* heterogeneity*. Cochran’s Q test verified the presence of heterogeneity and I^2^ test measured its statistical levels between studies.*

5. Line 24: Meta-regression is not an approach for identifying potential sources of heterogeneity

***Response*:** *Many thanks for your comment. We have removed the meta-regression analysis in our study.*

6. Line 29-30 It seems the sex disparity does not reach the significant difference

***Response*:** *Many thanks. The between-group difference was significant (Table 2)*

6. Line 31-32: How about the results of CD4 level in meta-regression? Please also provide the CI for the results.

***Response*:** *Many thanks for your comment. We have revised this part and removed the meta-regression analysis.*

7. The content of the introduction is a bit biased. Initiation of ART in the setting of an acute, AIDS-associated opportunistic infection does not follow the "the earlier the better" rule. For some opportunistic infections, such as cryptococcal and TB meningitis, immediate ART initiation may increase the risk of serious Concerns regarding immune reconstitution inflammatory syndrome. A short delay before initiating ART may be warranted. For patients with mild to moderate cutaneous Kaposi sarcoma, initiation of ART can not be done with chemotherapy. Drug interactions should be considered when starting ART.

***Response*:** *Many thanks. There is no doubt that what you said is true. However, according to the WHO guideline patients infected with HIV should start ART as soon as HIV diagnosis is confirmed including PLWH with* AIDS-associated opportunistic infection. And we have revised our discussion according to your comment.

8. Therefore, pooling the numbers of delayed ART initiation may not guide the clinical practice. The justification for the significance of this review is not clear enough.

***Response*:** *Many thanks for your comment. We thought that although there are some reasonable grounds to individualize treatment initiation to suit the patient’s overall health, initiating ART as soon as possible may be a key strategy in reaching the “95-95-95” goal. Our study may provide evidence relating to rapid-start ART. We also have revised this part.*

10. Line 100 The term "systematic review" and "meta-analysis" are used interchangeably.

***Response*:** *Many thanks for your comment. We have revised this part (Line 100)*

11. Line 100 Do You mean non-randomized studies? why does this review exclude randomized studies?

***Response*:** *Many thanks for your comment. We have revised this part (Lines 100,101). The articles reporting prevalence were predominantly observational studies, and the inclusion of RCT studies would increase the heterogeneity of the findings.*

12. Line 101 why does this review include minors?

***Response*:** *Many thanks. There are differences in the age division of adults globally. Some countries define adults as 15 years of age or older. In addition, studies with participants below the age of 15 years were excluded from our analysis due to their differences in HIV natural history from adults.*

13. Line 129-133 Please provide the statistical details of meta-regression and sensitivity analysis.

***Response*:** *Many thanks for your comment. We have revised this part (Line 134).*

14. Figure 1 The authors stated this review complies with PRISMA 2020. However, the PRISMA Flowchart did not fit the updated PRISMA.

***Response*:** *Many thankst. We have revised our Figure1.*

15. Table 1 Please provide only the surname of the first authors.

***Response*:** *Many thanks . We have revised this part (Table 1)*.

16. Table 1 Please provide the details of participants' characteristics including mean/median age, age range, sex, study setting, etc.

***Response*:** *Many thanks for your comment. We have added coutry income and sex (Table 1)*.

17. Table 1 Ross J et al Please provide the reasons in the footnote why this study did not report the quality score

***Response*:** *Many thanks for your comment. We have added the footnote below Table 1 (Line 161).*

17. Table 2 P for the difference in subgroups is needed

***Response*:** *Many thanks for your comment. We have added P in Table 2*.

18. Table 2 What do you mean by male and female subgroups? only including male/female? or the majority proportion?

***Response*:** *Many thank. Only including male/female.*

19. Table 2 mean age or median age? Do included studies report median age?

***Response*:** *Many thank. The age subgroup in this study was neither the mean nor the median age. Some of the original articles provide the number of delayed ART initiation at different age groups.*

20. Table 2 mean CD4 or median CD4?

***Response*:** *Many thank. The CD4 cell count subgroup in this study was neither the mean nor the median age. Some of the original articles provide the number of delayed ART initiation at different CD4 cell count groups.*

22. Table 3 It seems the authors misunderstand the difference between meta-regression and subgroup analysis. Year of publication, continent, country, country income, study design, sample size, and quality score could not be the covariates of prevalence of delayed ART initiation. Meta-regression usually included individual-level variables in regression models in each study.

***Response*:** *Many thanks for your comment. Due to the lack of original articles providing individual-level variables, we decided not to do meta-regression in this study, but only subgroup analysis. We have modified this part.*

23. Result what are the confounding factors set in the NOS?

***Response*:** *Many thanks.* Marks were given based on whether the original article was right about important confounding factors.

24. Did the authors search grey literature?

***Response*:** *Many thanks. We did not search grey literature and listed this as a limitation.*

25. Discussion: As I mentioned above, the delayed use of antiretroviral agents highly relies on the type of OI. This info should be mentioned in the discussion. Considerations when initiating ART are complicated.

***Response*:** *Many thanks. We have added this part (Line 210-217)*

26. The heterogeneity was high in all models. It should be listed as a limitation

***Response*:** *Many thanks for your comment. We have revised this part*.

Reviewer #2: General Comment:

This manuscript reported a systematic review studying the prevalence of delayed treatment (ART) to AIDS and its relevant factors. Overall, this paper contributed important knowledge to the literature. To improve the quality of the manuscript, I listed some suggestions below.

Major Comments:

1. Line 100. “No-randomized” is confusing. Did the study only include non-randomized studies?

***Response*:** *Many thanks. We have changed “non-random” to “cohort and cross-sectional study” (Line 100)*. The articles reporting prevalence were predominantly observational studies, and inclusion of RCT studies would increase the heterogeneity of the findings.

2. Line 101. Why did the authors only include studies published between January 2015 and August 2022?

***Response*:** *Many thanks. We have included only studies published since 2015, because the World Health Organization (WHO) recommends that all HIV-infected patients start ART immediately after HIV diagnosis is confirmed, regardless of the number of CD4 cells in 2015.*

2. Line 103-105. I suggest that the authors provide the reason(s) of excluding studies conducted among pregnant women.

***Response*:** *Many thanks for your comment.We have provided the reason of excluding studies conducted among pregnant women (Line 105, 106).*

3. Line 108-109. The two authors’ (Tao and Xie) reviewing process was not clearly described. For example, what were the “remaining relevant articles”?

***Response*:** *Many thanks. We have revised this part (Line 109-112)*.

5. The clarity of the process of screening titles and abstracts needs to be improved. For example, in Figure 1. “Record screened by title and abstract” appeared twice.

***Response*:** *Many thanks. We have revised the Figure 1*.

6. Line 153. I suggest that the authors explain the quality scores. For instance, what does the score of 3 mean?

***Response*:** *Many thanks. We have edded this (Line 123)*.

7. The resolution of figure 2 needs to be increased. The current version is difficult to read.

***Response*:** *Many thanks. We have revised the version of the Figure 2*.

8. The line 22 on page 17. The first rationale mentioned in the sentence – “the possible reason for the age difference is…for longer” – is difficult to understand. Also, the second rationale – “In addition, they usually…for young HIV-infected people” – is not convincing and needs to be rephrased.

***Response*:** *Many thanks. We have revised this sentence (Line 240-243).*

9. The line 9-10 on page 19. The second limitation was difficult to understand. Its clarity needs to be improved.

***Response*:** *Many thanks for your comment. We have revised this sentence (Line 268-275).*

Minor Comments:

1. Line 55. Please name the countries where the observational studies were conducted.

***Response*:** *Many thanks for your comment. We have revised this part (Line 53).*

2. Line 73. There is a grammar error. “Such time from…” needs to be revised to “such as time from…”

***Response*:** *Many thanks. We have added “as”.*

3. The manuscript will be strengthened, if the transition between the 3rd and 4th paragraphs could be improved.

***Response*:** *Many thanks for your comment. We have revised this part (Line 60-61).*

4. Line 83. The sentence “…global prevalence of delayed initiation for the time from diagnosis to ART initiation…” is difficult to understand. I suggest rephrasing this sentence.

***Response*:** *Many thanks. We have revised sentence (LIne 83,84).*

5. Line 145. I suggest deleting the word “subjects”.

***Response*:** *Many thanks. We have removed this word.*

6. In the section of subgroup analysis, the authors mentioned “high-income countries”, “upper-middle income countries”, and “lower-middle income countries”. I suggest adding a column in Table 1 to show the category of each country.

***Response*:** *Many thanks for your comment. We have added a column in table 1*.

7. Page 17. Some interventions mentioned in the discussion section were not clear. For example, what specific interventions were taken to streamline initiation of ART? What was the specific content of the “grassroots prevention and control service model”?

***Response*:** *Many thanks. We have interpreted those (Line 223-226).*

8. In the section of acknowledgements, the authors state that they thank all colleagues working in Xiangya Nursing School. This section will make more sense if the authors could briefly provide 1 or 2 reasons to illustrate why all colleagues need to be acknowledged.

***Response*:** *Many thanks. We have revised this part and removed this sentence.*

Again, thank you Editor, Dr. Chuanyi Ning, and reviewers. We are grateful for your consideration and for the potential opportunity to publish this paper in PLOS ONE.
