# Supplementary material for: Prevalence of delayed antiretroviral therapy initiation among people living with HIV: A systematic review and meta-analysis
Source: PLoS One. 2023 Oct 24;18(10):e0286476. doi: 10.1371/journal.pone.0286476 (PMC10597480; doi:10.1371/journal.pone.0286476)
Supplement: Peer review history

# Peer review: Decision Letter 0

**DOI:** 10.1371/journal.pone.0286476.r001

**Authors:** Chuanyi Ning

19 Dec 2022

PONE-D-22-26544Prevalence of delayed antiretroviral therapy initiation among people living with HIV: a systematic review and meta-analysisPLOS ONE

Dear Dr. Wang,

Thank you for submitting your manuscript to PLOS ONE. After careful consideration, we feel that it has merit but does not fully meet PLOS ONE’s publication criteria as it currently stands. Therefore, we invite you to submit a revised version of the manuscript that addresses the points raised during the review process.

Please submit your revised manuscript by Feb 02 2023 11:59PM. If you will need more time than this to complete your revisions, please reply to this message or contact the journal office at plosone@plos.org. When you're ready to submit your revision, log on to https://www.editorialmanager.com/pone/ and select the 'Submissions Needing Revision' folder to locate your manuscript file.

Please include the following items when submitting your revised manuscript:

- A rebuttal letter that responds to each point raised by the academic editor and reviewer(s). You should upload this letter as a separate file labeled 'Response to Reviewers'.
- A marked-up copy of your manuscript that highlights changes made to the original version. You should upload this as a separate file labeled 'Revised Manuscript with Track Changes'.
- An unmarked version of your revised paper without tracked changes. You should upload this as a separate file labeled 'Manuscript'.

If you would like to make changes to your financial disclosure, please include your updated statement in your cover letter. Guidelines for resubmitting your figure files are available below the reviewer comments at the end of this letter.

If applicable, we recommend that you deposit your laboratory protocols in protocols.io to enhance the reproducibility of your results. Protocols.io assigns your protocol its own identifier (DOI) so that it can be cited independently in the future. For instructions see: https://journals.plos.org/plosone/s/submission-guidelines#loc-laboratory-protocols. Additionally, PLOS ONE offers an option for publishing peer-reviewed Lab Protocol articles, which describe protocols hosted on protocols.io. Read more information on sharing protocols at https://plos.org/protocols?utm_medium=editorial-email&utm_source=authorletters&utm_campaign=protocols.

We look forward to receiving your revised manuscript.

Kind regards,

Chuanyi Ning, Ph.D.

Academic Editor

PLOS ONE

Journal Requirements:

When submitting your revision, we need you to address these additional requirements.

1. Please ensure that your manuscript meets PLOS ONE's style requirements, including those for file naming. The PLOS ONE style templates can be found at 

https://journals.plos.org/plosone/s/file?id=wjVg/PLOSOne_formatting_sample_main_body.pdf and 

https://journals.plos.org/plosone/s/file?id=ba62/PLOSOne_formatting_sample_title_authors_affiliations.pdf

[Note: HTML markup is below. Please do not edit.]

Reviewers' comments:

Reviewer's Responses to Questions

**Comments to the Author**

1. Is the manuscript technically sound, and do the data support the conclusions?

The manuscript must describe a technically sound piece of scientific research with data that supports the conclusions. Experiments must have been conducted rigorously, with appropriate controls, replication, and sample sizes. The conclusions must be drawn appropriately based on the data presented. 

Reviewer #1: Partly

Reviewer #2: Yes

**********

2. Has the statistical analysis been performed appropriately and rigorously? 

Reviewer #1: Yes

Reviewer #2: I Don't Know

**********

3. Have the authors made all data underlying the findings in their manuscript fully available?

The PLOS Data policy requires authors to make all data underlying the findings described in their manuscript fully available without restriction, with rare exception (please refer to the Data Availability Statement in the manuscript PDF file). The data should be provided as part of the manuscript or its supporting information, or deposited to a public repository. For example, in addition to summary statistics, the data points behind means, medians and variance measures should be available. If there are restrictions on publicly sharing data—e.g. participant privacy or use of data from a third party—those must be specified.

Reviewer #1: Yes

Reviewer #2: Yes

**********

4. Is the manuscript presented in an intelligible fashion and written in standard English?

PLOS ONE does not copyedit accepted manuscripts, so the language in submitted articles must be clear, correct, and unambiguous. Any typographical or grammatical errors should be corrected at revision, so please note any specific errors here.

Reviewer #1: Yes

Reviewer #2: Yes

**********

5. Review Comments to the Author

Please use the space provided to explain your answers to the questions above. You may also include additional comments for the author, including concerns about dual publication, research ethics, or publication ethics. (Please upload your review as an attachment if it exceeds 20,000 characters)

Reviewer #1: Please consider the following comments:

1. The manuscript should be proofread carefully.

2. Line 15: Please delete the working definition for delayed antiretroviral therapy

3. Line 21: Please provide both names of the database and platform (e.g. Pubmed/Medline)

4. Line 23: Why the authors used both approaches for detecting heterogeneity?

5. Line 24: Meta-regression is not an approach for identifying potential sources of heterogeneity

6. Line 29-30 It seems the sex disparity does not reach the significant difference

7. Line 31-32: How about the results of CD4 level in meta-regression? Please also provide the CI for the results.

8. The content of the introduction is a bit biased. Initiation of ART in the setting of an acute, AIDS-associated opportunistic infection does not follow the "the earlier the better" rule. For some opportunistic infections, such as cryptococcal and TB meningitis, immediate ART initiation may increase the risk of serious Concerns regarding immune reconstitution inflammatory syndrome. A short delay before initiating ART may be warranted. For patients with mild to moderate cutaneous Kaposi sarcoma, initiation of ART can not be done with chemotherapy. Drug interactions should be considered when starting ART.

9. Therefore, pooling the numbers of delayed ART initiation may not guide the clinical practice. The justification for the significance of this review is not clear enough.

10. Line 100 The term "systematic review" and "meta-analysis" are used interchangeably.

11. Line 100 Do You mean non-randomized studies? why does this review exclude randomized studies?

12. Line 101 why does this review include minors?

13. Line 129-133 Please provide the statistical details of meta-regression and sensitivity analysis.

14. Figure 1 The authors stated this review complies with PRISMA 2020. However, the PRISMA Flowchart did not fit the updated PRISMA.

15. Table 1 Please provide only the surname of the first authors.

16. Table 1 Please provide the details of participants' characteristics including mean/median age, age range, sex, study setting, etc.

17. Table 1 Ross J et al Please provide the reasons in the footnote why this study did not report the quality score

18. Table 2 P for the difference in subgroups is needed

19. Table 2 What do you mean by male and female subgroups? only including male/female? or the majority proportion?

20. Table 2 mean age or median age? Do included studies report median age?

21. Table 2 mean CD4 or median CD4?

22. Table 3 It seems the authors misunderstand the difference between meta-regression and subgroup analysis. Year of publication, continent, country, country income, study design, sample size, and quality score could not be the covariates of prevalence of delayed ART initiation. Meta-regression usually included individual-level variables in regression models in each study.

23. Result what are the confounding factors set in the NOS?

24. Did the authors search grey literature?

25. Discussion: As I mentioned above, the delayed use of antiretroviral agents highly relies on the type of OI. This info should be mentioned in the discussion. Considerations when initiating ART are complicated.

26. The heterogeneity was high in all models. It should be listed as a limitation

Reviewer #2: General Comment:

This manuscript reported a systematic review studying the prevalence of delayed treatment (ART) to AIDS and its relevant factors. Overall, this paper contributed important knowledge to the literature. To improve the quality of the manuscript, I listed some suggestions below.

Major Comments:

1. Line 100. “No-randomized” is confusing. Did the study only include non-randomized studies?

2. Line 101. Why did the authors only include studies published between January 2015 and August 2022?

3. Line 103-105. I suggest that the authors provide the reason(s) of excluding studies conducted among pregnant women.

4. Line 108-109. The two authors’ (Tao and Xie) reviewing process was not clearly described. For example, what were the “remaining relevant articles”?

5. The clarity of the process of screening titles and abstracts needs to be improved. For example, in Figure 1. “Record screened by title and abstract” appeared twice.

6. Line 153. I suggest that the authors explain the quality scores. For instance, what does the score of 3 mean?

7. The resolution of figure 2 needs to be increased. The current version is difficult to read.

8. The line 22 on page 17. The first rationale mentioned in the sentence – “the possible reason for the age difference is…for longer” – is difficult to understand. Also, the second rationale – “In addition, they usually…for young HIV-infected people” – is not convincing and needs to be rephrased.

9. The line 9-10 on page 19. The second limitation was difficult to understand. Its clarity needs to be improved.

Minor Comments:

1. Line 55. Please name the countries where the observational studies were conducted.

2. Line 73. There is a grammar error. “Such time from…” needs to be revised to “such as time from…”

3. The manuscript will be strengthened, if the transition between the 3rd and 4th paragraphs could be improved.

4. Line 83. The sentence “…global prevalence of delayed initiation for the time from diagnosis to ART initiation…” is difficult to understand. I suggest rephrasing this sentence.

5. Line 145. I suggest deleting the word “subjects”.

6. In the section of subgroup analysis, the authors mentioned “high-income countries”, “upper-middle income countries”, and “lower-middle income countries”. I suggest adding a column in Table 1 to show the category of each country.

7. Page 17. Some interventions mentioned in the discussion section were not clear. For example, what specific interventions were taken to streamline initiation of ART? What was the specific content of the “grassroots prevention and control service model”?

8. In the section of acknowledgements, the authors state that they thank all colleagues working in Xiangya Nursing School. This section will make more sense if the authors could briefly provide 1 or 2 reasons to illustrate why all colleagues need to be acknowledged.

**********

6. PLOS authors have the option to publish the peer review history of their article (what does this mean?). If published, this will include your full peer review and any attached files.

If you choose “no”, your identity will remain anonymous but your review may still be made public.

**Do you want your identity to be public for this peer review?** For information about this choice, including consent withdrawal, please see our Privacy Policy.

Reviewer #1: No

Reviewer #2: **Yes: **Zhao Ni

**********

[NOTE: If reviewer comments were submitted as an attachment file, they will be attached to this email and accessible via the submission site. Please log into your account, locate the manuscript record, and check for the action link "View Attachments". If this link does not appear, there are no attachment files.]

While revising your submission, please upload your figure files to the Preflight Analysis and Conversion Engine (PACE) digital diagnostic tool, https://pacev2.apexcovantage.com/. PACE helps ensure that figures meet PLOS requirements. To use PACE, you must first register as a user. Registration is free. Then, login and navigate to the UPLOAD tab, where you will find detailed instructions on how to use the tool. If you encounter any issues or have any questions when using PACE, please email PLOS at figures@plos.org. Please note that Supporting Information files do not need this step.

# Author comment: Author response to Decision Letter 0

**DOI:** 10.1371/journal.pone.0286476.r002

2 Feb 2023

Dear Editor and Reviewers

Thank you for reviewing our manuscript PONE-D-22-26544 “Prevalence of delayed antiretroviral therapy initiation among people living with HIV: a systematic review and meta-analysis”. We found the comments very helpful in improving our manuscript. In the document "response to reviewers", we list the reviewer comments and our responses. We had presented the updated changes in “italic”. 

Again, thank you Editor and reviewers. We are grateful for your consideration and for the potential opportunity to publish this paper in PLOS ONE.

Attachment Submitted filename: Response to reviwers.docx Click here for additional data file.

# Peer review: Decision Letter 1

**DOI:** 10.1371/journal.pone.0286476.r003

**Authors:** Lara Vojnov

17 May 2023

Prevalence of delayed antiretroviral therapy initiation among people living with HIV: a systematic review and meta-analysis

PONE-D-22-26544R1

Dear Dr. Wang,

We’re pleased to inform you that your manuscript has been judged scientifically suitable for publication and will be formally accepted for publication once it meets all outstanding technical requirements.

Within one week, you’ll receive an e-mail detailing the required amendments. When these have been addressed, you’ll receive a formal acceptance letter and your manuscript will be scheduled for publication.

An invoice for payment will follow shortly after the formal acceptance. To ensure an efficient process, please log into Editorial Manager at http://www.editorialmanager.com/pone/, click the 'Update My Information' link at the top of the page, and double check that your user information is up-to-date. If you have any billing related questions, please contact our Author Billing department directly at authorbilling@plos.org.

If your institution or institutions have a press office, please notify them about your upcoming paper to help maximize its impact. If they’ll be preparing press materials, please inform our press team as soon as possible -- no later than 48 hours after receiving the formal acceptance. Your manuscript will remain under strict press embargo until 2 pm Eastern Time on the date of publication. For more information, please contact onepress@plos.org.

Kind regards,

Lara Vojnov

Academic Editor

PLOS ONE

Additional Editor Comments (optional):

Reviewers' comments:

Reviewer's Responses to Questions

**Comments to the Author**

1. If the authors have adequately addressed your comments raised in a previous round of review and you feel that this manuscript is now acceptable for publication, you may indicate that here to bypass the “Comments to the Author” section, enter your conflict of interest statement in the “Confidential to Editor” section, and submit your "Accept" recommendation.

Reviewer #1: All comments have been addressed

Reviewer #2: All comments have been addressed

**********

2. Is the manuscript technically sound, and do the data support the conclusions?

The manuscript must describe a technically sound piece of scientific research with data that supports the conclusions. Experiments must have been conducted rigorously, with appropriate controls, replication, and sample sizes. The conclusions must be drawn appropriately based on the data presented. 

Reviewer #1: Yes

Reviewer #2: Yes

**********

3. Has the statistical analysis been performed appropriately and rigorously? 

Reviewer #1: Yes

Reviewer #2: I Don't Know

**********

4. Have the authors made all data underlying the findings in their manuscript fully available?

The PLOS Data policy requires authors to make all data underlying the findings described in their manuscript fully available without restriction, with rare exception (please refer to the Data Availability Statement in the manuscript PDF file). The data should be provided as part of the manuscript or its supporting information, or deposited to a public repository. For example, in addition to summary statistics, the data points behind means, medians and variance measures should be available. If there are restrictions on publicly sharing data—e.g. participant privacy or use of data from a third party—those must be specified.

Reviewer #1: Yes

Reviewer #2: Yes

**********

5. Is the manuscript presented in an intelligible fashion and written in standard English?

PLOS ONE does not copyedit accepted manuscripts, so the language in submitted articles must be clear, correct, and unambiguous. Any typographical or grammatical errors should be corrected at revision, so please note any specific errors here.

Reviewer #1: Yes

Reviewer #2: No

**********

6. Review Comments to the Author

Please use the space provided to explain your answers to the questions above. You may also include additional comments for the author, including concerns about dual publication, research ethics, or publication ethics. (Please upload your review as an attachment if it exceeds 20,000 characters)

Reviewer #1: I am satisfied with the revisions made to the manuscript. Thank you for your diligent efforts in addressing the concerns and improving the overall quality of the document.

Reviewer #2: The authors addressed my comments. There are still some grammatical errors in the manuscript. For example, "clinical management of these patients beingcomplex." should be revised to "clinical management of these patients is complex."

**********

7. PLOS authors have the option to publish the peer review history of their article (what does this mean?). If published, this will include your full peer review and any attached files.

If you choose “no”, your identity will remain anonymous but your review may still be made public.

**Do you want your identity to be public for this peer review?** For information about this choice, including consent withdrawal, please see our Privacy Policy.

Reviewer #1: No

Reviewer #2: No

**********

# Editor report: Acceptance letter

**DOI:** 10.1371/journal.pone.0286476.r004

**Authors:** Lara Vojnov

23 May 2023

PONE-D-22-26544R1 

Prevalence of delayed antiretroviral therapy initiation among people living with HIV: a systematic review and meta-analysis 

Dear Dr. Wang:

I'm pleased to inform you that your manuscript has been deemed suitable for publication in PLOS ONE. Congratulations! Your manuscript is now with our production department. 

If your institution or institutions have a press office, please let them know about your upcoming paper now to help maximize its impact. If they'll be preparing press materials, please inform our press team within the next 48 hours. Your manuscript will remain under strict press embargo until 2 pm Eastern Time on the date of publication. For more information please contact onepress@plos.org.

If we can help with anything else, please email us at plosone@plos.org. 

Thank you for submitting your work to PLOS ONE and supporting open access. 

Kind regards, 

PLOS ONE Editorial Office Staff

on behalf of

Dr. Lara Vojnov 

Academic Editor

PLOS ONE
